# A Severe Case of Drug-Induced Liver Injury after Gemcitabine Administration: A Highly Probable Causality Grading as Assessed by the Updated RUCAM Diagnostic Scoring System

**DOI:** 10.1155/2020/8812983

**Published:** 2020-10-01

**Authors:** Ilenia Mascherona, Caterina Maggioli, Maira Biggiogero, Oreste Mora, Lucia Marelli

**Affiliations:** ^1^Ospedale La Carità, Medicina Interna, Locarno, Switzerland; ^2^AUSL di Bologna, Ospedale di Budrio, Bologna, Italy; ^3^Clinica Luganese Moncucco, Unità di Ricerca Clinica, Lugano, Switzerland; ^4^Clinica Luganese Moncucco, Oncologia Medica, Lugano, Switzerland

## Abstract

Gemcitabine is an antineoplastic drug used in several forms of advanced pancreatic, lung, breast, ovarian, and bladder cancer. Common side effects include bone marrow suppression, fatigue, diarrhea, nausea, gastrointestinal upset, rash, alopecia, and stomatitis. Transient serum enzyme elevations could be observed during therapy, but clinically significant acute liver injury has been rarely associated with its use. Few cases of acute liver injury have been reported in the literature. We reported the clinical case of a 73--year-old man who developed clinically significant acute hepatic injury after using gemcitabine. Possible causes, clinical presentation, and treatments are discussed. According to the updated RUCAM score, the case was rated 10 points and became a suspected drug-induced liver injury. Moreover, on the liver biopsy, there were histological findings of mild-to-moderate portal hepatitis, eosinophilia, bile duct injury, and mild perisinusoidal fibrosis, suggesting drug damage.

## 1. Introduction

Gemcitabine is a cytosine analog [1, 2] which is used as an antineoplastic drug in the therapy of several forms of advanced pancreatic, lung, breast, ovarian, and bladder cancer. Common side effects include bone marrow suppression, fatigue, flu-like symptoms, diarrhea, nausea, gastrointestinal upset, and stomatitis.

Gemcitabine could be associated with transient serum enzyme elevations during therapy, but it is rarely a cause of acute, clinically apparent liver injury [3, 4]. Elevations in serum aminotransferase levels occur in 30% to 90% of patients [5–7]. These elevations are generally mild to moderate [8], asymptomatic, and self-limited, frequently resolving without discontinuation of the therapy. Serum bilirubin and alkaline phosphatase elevations are less common, but typically transient and mild. Few cases of acute hepatic injury due to gemcitabine have been reported. The clinical features of gemcitabine hepatotoxicity are not well described. Gemcitabine modification or discontinuation is considered only in drug-induced liver injury (DILI) cases.

We describe the case of a 73-year-old man who developed a DILI after gemcitabine treatment. A few DILI cases due to gemcitabine administration have been reported, most of them in patients with underlying chronic liver disease or extensive hepatic metastases [9–11].

Patient demographic data, details of diagnosis and treatment, and laboratory and radiological investigations were collected by the DPI electronic database of Clinica Luganese Moncucco (CLM), Lugano. The patient gave his informed consent before any data were entered into the database.

## 2. Case Presentation

On 21st September 2018, a 73-year-old man was admitted in the Department of Oncology of CLM for jaundice, itching, acholic feces, and hyperchromic urine. He was recently diagnosed with an urothelial carcinoma and treated with neoadjuvant chemotherapy (carboplatin and gemcitabine). One week before the admission, the patient reported an episode of diarrhea after eating cooked fish. The patient denied alcohol consumption and psychotropic drugs and herbal product assumption. He took his usual drug therapy (described in the following) and no other drugs.

On physical examination, the patient was alert, oriented to person, time, and place. Vital signs were normal, and body temperature was normal. The cutaneous examination revealed jaundice. No jugular turgescence, peripheral edema, flapping tremor, or hepatosplenomegaly was found. The rest of the clinical examination was without any particularities.

The patient's medical history reported benign prostatic hyperplasia, chronic gastritis, diverticulosis, right glaucoma, and iodinated contrast urticaria. His usual therapy was pantoprazole (40 mg/d), tamsulosin (400 mcg/d), and timolol (1 drop 2 times a day). On July 2018, a papillary urothelial carcinoma was diagnosed. This cancer infiltrated the bladder muscolaris propria, caused a perineural invasion, and was poorly differentiated (stage pT2N0M0, G3).

On 10th of August 2018, oncologists indicated neoadjuvant chemotherapy with carboplatin 5 AUC d1 and gemcitabine 1000 mg/sqm d1 and d8 every 21 days. Carboplatin was administered on the 10th and 31st of August, and gemcitabine was administered on the 10th, 17th, and 31st of August.

At admission, laboratory findings showed normocytic normochromic anemia (113 g/l); white blood cells and platelets were within normal limits. High hyperbilirubinemia (6.37 mg/dl) with a prevalence of conjugated bilirubin (5.96 mg/dl) and hypertransaminasemia (AST 82 U/l and ALT 158 U/l) were observed/found. Cholestasis enzymes were increased (ALP 407 U/l and GGT 186 U/l), and the ammonia level was high (134.54 ug/dl). Albumin level was within normal limits. No coagulation, renal, or electrolyte abnormalities were found (see Table 1).

A differential diagnosis of hepatitis was quickly started. Viral (HAV, HBV, HCV, HDV, HEV, CMV, and EBV) and autoimmune (ANA, ENA, ANCA, ASMA, AMA, anti-SLA/LP, anti-LC1, and anti-LKM immunoglobulins) hepatitis serology was performed with negative results. The celiac disease profile was negative. Alpha-1-antitrypsin, ceruloplasmin, carbohydrate-deficient transferrin (CDT), IgA, and alpha-fetoprotein (AFP) were normal. An abdominal CT scan and a magnetic resonance cholangiopancreatography (MRCP) were performed showing only an hepatic hemangioma of the VIII segment.

During the hospitalization, a symptomatic therapy with levocetirizine and dexamethasone was successfully carried out. On 24th September 2018, the patient was discharged with mild symptoms and improved laboratory findings (see Table 1).

**Figure 1:**
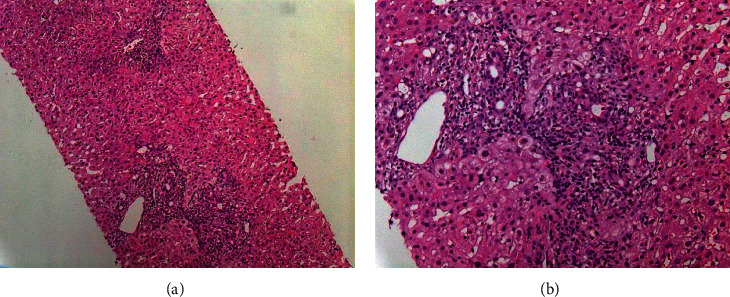
(a) Performed liver biopsy which showed mild-to-moderate portal hepatitis with interface component, minimal lobular component, eosinophilia, bile duct injury, and mild perisinusoidal fibrosis. (b) Magnification.

No etiology was found, and an adverse drug reaction (ADR) was supposed. Hepatology consultation was performed, and chemotherapy (especially gemcitabine) was taken into account as a hepatitis cause. Gemcitabine was interrupted, and the patient underwent a liver biopsy. It suggested a drug injury, characterized by mild-to-moderate portal hepatitis, eosinophilia, bile duct injury, and mild perisinusoidal fibrosis (see Figure 1).

One month later, the patient was re-evaluated on an oncological check-up and showed a good health condition. There was no jaundice; urine and stools were normalized, and pruritus was resolved. Liver values were completely normalized, and an abdomen CT scan confirmed the absence of abnormalities.

## 3. Discussion

Drug-induced liver injury (DILI) is a common liver disease which generally occurs between several days and few months after drug ingestion [12]. It is a common side effect that often leads pharmaceutical companies to withdraw the responsible drug from the market [13]. Its prevalence is between 1 and 20 in 100,000 or less in developed countries [14]. Several risk factors are associated with the development of DILI [15]. For instance, it has been shown that adults are more at risk than children and women who seem to be more susceptible than men. Potentially, all drugs can be involved in DILI development, but it is more commonly experienced with acetaminophen [16, 17], antibiotics [18], NSAIDS, statins, antiplatelets, and immunosuppressant administration.

The clinical manifestations of DILI are usually nonspecific. The majority of patients manifests liver biochemical index abnormalities. Some patients might express unspecific symptoms such as fatigue, low-grade fever, anorexia, nausea, vomiting, liver pain, and epigastric discomfort. Besides, the patient with obvious cholestasis could manifest jaundice, itching, acholic stools, and hyperchromic urine. Rarely, the patient can experience hepatic failure. Hepatomegaly may be present on physical examination. In severe cases, coagulopathy and hepatic encephalopathy may develop, indicating acute liver failure. In acute DILI, the liver indexes are impaired for less than three months. In chronic DILI, instead, liver values remain altered for more than three months [19]. Chronic DILI may lead to fibrosis or cirrhosis and could have signs and symptoms associated with cirrhosis or hepatic decompensation [20–23]. The presence of jaundice, a double value of bilirubinemia, and an increase in transaminases are associated with a worse prognosis [24].

According to the pathogenesis, DILI can be classified into 2 types: intrinsic and idiosyncratic. The latter is more frequent, is not related to drug dosage, and has variable latency periods. The hypothesized mechanisms underlying hepatotoxicity include cell membrane destruction and cell death, development of an immunological reaction, inhibition of cellular metabolism pathways, abnormal bile flow, apoptosis, and inhibition of mitochondrial function.

According to the pattern of observed liver tests, there are 3 types of DILI: hepatocellular (hepatitis damage), cholestatic (biliary tract damage), and mixed, which was proposed by the Roussel Uclaf Causality Assessment Method (RUCAM) [12, 25]. DILI cholestasis is defined as an alanine aminotransferase (ALT) to alkaline phosphatase (ALP) ratio of less than 2. Injury is considered to be mixed if the ALT/ALP ratio is greater than 2 but less than 5 and hepatocellular if this ratio is >5.

Since there are no diagnostic tests or specific biomarkers for DILI, its diagnosis is made by exclusion of other causes of liver disease, including viral and autoimmune hepatitis, bile duct obstruction, hepatic ischemia, sepsis, and metabolic disorders. The updated RUCAM diagnostic criteria [12, 25] are also helpful: it stratifies the causal correlation between drugs and liver injury into 4 levels, high probable: score > 8, probable: 6 ≤ score ≤ 8, possible: 3 ≤ score ≤, and unlikely: 1 ≤ score ≤ 2. Combined with these criteria, the diagnosis of DILI must rely on medical anamnesis and all clinical data. When necessary, DILI could be confirmed by liver biopsy.

If DILI is suspected, the primary treatment is the withdrawal of the offending drug. Thereafter, the patient should be treated on a symptomatic level. After the drug removal, the majority of patients clearly improved. 5 to 10% of cholestatic/mixed types of DILI, instead, progress to chronic disease [26].

DILI patients should be followed up with serial biochemical measurements until complete liver test resolution. Hepatology consultation should be considered in front of acute liver failure.

In our case report, the patient was a 73-year-old man who developed hepatitis with jaundice. He was treated with gemcitabine 21 days before his admission to CLM. Viral and autoimmune hepatitis and other hepatitis etiologies were excluded. The updated RUCAM scoring [12, 20, 25] was done, the patient was rated 10 points, and for this reason, our case had a high probability of being a DILI, a mixed-type DILI to be exact (ALT/ALP ratio of 2.75). The liver biopsy and the improvement in symptoms and liver values after gemcitabine discontinuation confirm the drug-induced liver injury.

Our case report evaluates gemcitabine inducing liver toxicity. Gemcitabine (2′,2′-difluoro-2′-deoxycytidine (dFdC)) is a deoxycytidine analog with multiple modes of action inside the cell. It is phosphorylated by deoxycytidine kinase to the active compounds gemcitabine di- and triphosphate. Gemcitabine diphosphate can be toxic if the pool of cellular ATP decreases. Its principal pharmacological activity is incorporated into DNA during replication in the S phase of the cell cycle, following the inactivation of DNA polymerases and the inhibition of DNA synthesis. Gemcitabine activity primarily consists of inducing cell cycle arrest and cell death. The induction of apoptosis through caspase signaling is also another important mechanism of action [27]. The precise molecular mechanisms determining tumor cell responses to gemcitabine and the impact of mechanistic interactions with other chemotherapeutic agents remain unelucidated [28]. Moreover, gemcitabine hepatic metabolism has its cornerstone into cytidine deaminase (CDA), encoded by the CDA gene located in locus 1p36.2–35 [29]. Several studies demonstrated that single-nucleotide polymorphism of CDA, resulting in decreased serum CDA concentration, may lead to severe toxicity induced by gemcitabine [30–32]. Usually, gemcitabine is well tolerated, with common side effects including nausea and vomiting, rash, fever, flu-like symptoms, and peripheral edema. Myelosuppression is the most common dose-limiting toxicity. Also, various pulmonary toxicities of gemcitabine have been reported [33]. Gemcitabine can cause a transient increase in liver indexes, whereas a more serious hepatic involvement up to fatal outcome is less frequent [7]. In our case report, in the absence of other causes, we believe that gemcitabine is the main cause of DILI. We do not consider carboplatin, concomitantly administered with gemcitabine [34, 35], as a DILI cause, due to very few cases of liver abnormalities in the literature, generally related to high dosages [36]. Discontinuation of gemcitabine, together with purely symptomatic therapy, lead us to a clinical resolution and normalization of laboratory indexes.

Moreover, comparing the case reports cited in [3, 4, 10, 11], our case report uses the RUCAM [12, 20, 25] diagnostic scoring system as a fundamental tool in the DILI diagnostic process.

## 4. Conclusion

In conclusion, clinicians should pay particular attention to the liver context, when starting a gemcitabine-based treatment regimen. A careful remote anamnesis and pharmacological history should be taken in order to identify risk factors that can contribute to the development of a drug-induced liver injury. Therefore, especially in follow-up care, clinicians should do thorough clinical and laboratory monitoring in order to promptly diagnose this rare adverse drug reaction.

## Figures and Tables

**Table 1:** Patient liver values from hospitalization up to the follow-up.

	23.10.18	11.10.18	03.10.18	28.09.18	24.09.18	23.09.18	22.09.18	21.09.18

Total bilirubin (<1.23 mg/dl)	1.12	2.02	3.64	7.49	6.67	6.08	6.37	6.78

Direct bilirubin (<0.29 mg/dl)	—	1.74	3.12	6.55	5.96	5.64	5.96	5.77

Aspartate aminotransferase (10–50 U/L)	24	61	100	154	94	88	82	76

Alanine transaminase (10–50 U/L)	46	158	329	325	182	167	158	161

Alkaline phosphatase (40–129 U/L)	108	199	292	347	381	385	407	444

Albumin (35–52 g/L)	40	36	36	36	34	—	—	37

## Data Availability

All the data are available in the patient's medical chart.

## Abbreviations

ADR: Adverse drug reaction
AFP: Alpha-fetoprotein
ALP: Alkaline phosphatase
ALT: Alanine aminotransferase
AMA: Antimitochondrial auto-antibodies
ANA: Antinuclear antibodies
ANCA: Antineutrophil cytoplasmic antibodies
Anti-LC1: Antiliver cytosolic antigen type 1
Anti-LKM: Liver/kidney microsomal antibodies
Anti-SLA/LP: Antisoluble liver antigen/liver-pancreas
ASMA: Anti-smooth muscle antibodies
AST: Aspartate aminotransferase
CDA: Cytidine deaminase
CDT: Carbohydrate-deficient transferrin
CLM: Clinica Luganese Moncucco
CMV: Cytomegalovirus
CT: Computed tomography
DILI: Drug-induced liver injury
EBV: Epstein–Barr virus
ENA: Extractable nuclear antigen
GGT: Gamma-glutamyl transferase
HAV: Hepatovirus A
HBV: Hepatovirus B
HCV: Hepatovirus C
HDV: Hepatovirus D
HEV: Hepatovirus E
MRCP: Magnetic resonance cholangiopancreatography
NSAIDS: Nonsteroidal anti-inflammatory drugs
RUCAM: Roussel Uclaf Causality Assessment Method.
